# Integrating GPT-4o Into Data Mining in Neurosurgery: Feasibility and Proof-of-Concept Study

**DOI:** 10.2196/77114

**Published:** 2026-03-09

**Authors:** Arthur Henrique Almeida Sales, Jürgen Beck, Jürgen Grauvogel

**Affiliations:** 1 Department of Neurosurgery, Faculty of Medicine, University of Freiburg, Breisacher Str. 64, Freiburg, 79106, Germany, 49 761-270 ext 50010

**Keywords:** artificial intelligence, AI, data mining, GPT-4o, patient privacy, automated coding, health care analytics

## Abstract

**Background:**

Large language models offer new possibilities for transforming unstructured clinical text into structured datasets. However, their performance in specialized and complex documentation environments, such as neurosurgery, remains insufficiently characterized. GPT-4o is a large language model with enhanced natural language capabilities, but its accuracy in extracting structured data from neurosurgical reports has not been systematically assessed.

**Objective:**

This proof-of-concept study evaluated the feasibility and accuracy of GPT-4o for extracting predefined structured variables from unstructured neurosurgical reports of patients with vestibular schwannoma. Specific aims were to measure accuracy across variable types, assess the impact of prompt refinement, and explore the model’s potential utility for research-oriented data mining.

**Methods:**

In this retrospective single-center study, 10 consecutive patients with histologically confirmed vestibular schwannoma who underwent surgery between August and December 2023 were included. Four anonymized German-language documents per patient (discharge, surgical, histopathology, and 3-month follow-up reports) were processed using GPT-4o. Seventeen variables were extracted using a standardized zero-shot prompt. Targeted prompt refinements were subsequently applied for variables with low baseline accuracy. Two board-certified neurosurgeons independently validated all outputs, with discrepancies resolved by a senior neurosurgeon. Accuracy metrics, 95% CIs (Wilson method), and descriptive comparisons between variable types were calculated.

**Results:**

GPT-4o achieved 100% accuracy for structured variables requiring minimal interpretation, including patient ID, date of birth, date of surgery, histopathological diagnosis, and World Health Organization grade. Several interpretative variables, such as symptoms at presentation, symptom type, symptom duration, extent of resection, and permanence of postoperative deficits, were also extracted with 100% accuracy. In contrast, intraoperative complications and new postoperative deficits were correctly identified in only 50% (5/10) of cases using the zero-shot prompt. After targeted prompt refinement, accuracy for these variables improved substantially, reaching 90% to 100% in most cases. The mean accuracy was highest for structured categorical variables (97.5%, SD 4.6%), intermediate for binary variables (80%, SD 27.4%), and lowest for conditional text variables (66.7%, SD 28.9%), without statistically significant differences (*P*=.25).

**Conclusions:**

GPT-4o demonstrated strong feasibility for structured data extraction from standardized neurosurgical reports, particularly for variables with limited semantic complexity. However, the high accuracy observed reflects a narrow and highly controlled context and should not be interpreted as evidence of general reliability across diverse clinical settings. Larger, multi-institutional, and multilingual studies are needed to determine broader applicability and potential clinical integration.

## Introduction

The advent of artificial intelligence (AI) in health care has revolutionized data processing, decision-making, and research methodologies. Among these innovations, natural language processing (NLP) models, such as GPT-4o (released in May 2024; OpenAI), represent a significant leap in handling unstructured data. With their capacity to synthesize, analyze, and structure information, large language models (LLMs) provide a promising avenue for transforming how clinicians and researchers interact with medical records and literature.

In neurosurgery, the challenge of efficiently extracting insights from diverse and voluminous data, including imaging reports, clinical notes, and surgical documentation, is particularly pronounced. Studies have demonstrated that NLP models can bridge these gaps, optimizing both clinical workflows and research efforts [1,2]. Furthermore, by automating labor-intensive processes, these tools may enhance patient care, reduce costs, and streamline health care operations [3].

Despite the promise, challenges remain. Privacy concerns, data security, and the need for precise tuning of AI tools pose significant barriers to their widespread adoption [4-6]. While LLMs present remarkable opportunities, their limitations must also be recognized. Common challenges include hallucination, biases inherited from training data, and overfitting in specialized domains. These issues have historically limited their practical use in health care, highlighting the need for thorough validation and responsible implementation.

This proof-of-concept study aimed to explore the application of GPT-4o as a data mining tool in neurosurgery. Patients with vestibular schwannoma were selected because their management involves standardized documentation, including surgical, pathological, and follow-up reports. This provides a consistent and well-defined framework for assessing the capability of GPT-4o to structure complex clinical data. The approach presented here may serve as a template for future applications across other neurosurgical conditions. Specifically, we seek to evaluate the model’s ability to extract and provide structured data from unstructured datasets and assess its feasibility as an aid in research environments.

By leveraging GPT-4’s NLP capabilities, we hypothesize that neurosurgical data can be extracted and structured reliably, facilitating accurate data aggregation and analysis across heterogeneous clinical reports.

## Methods

### Overview

**Figure 1.**
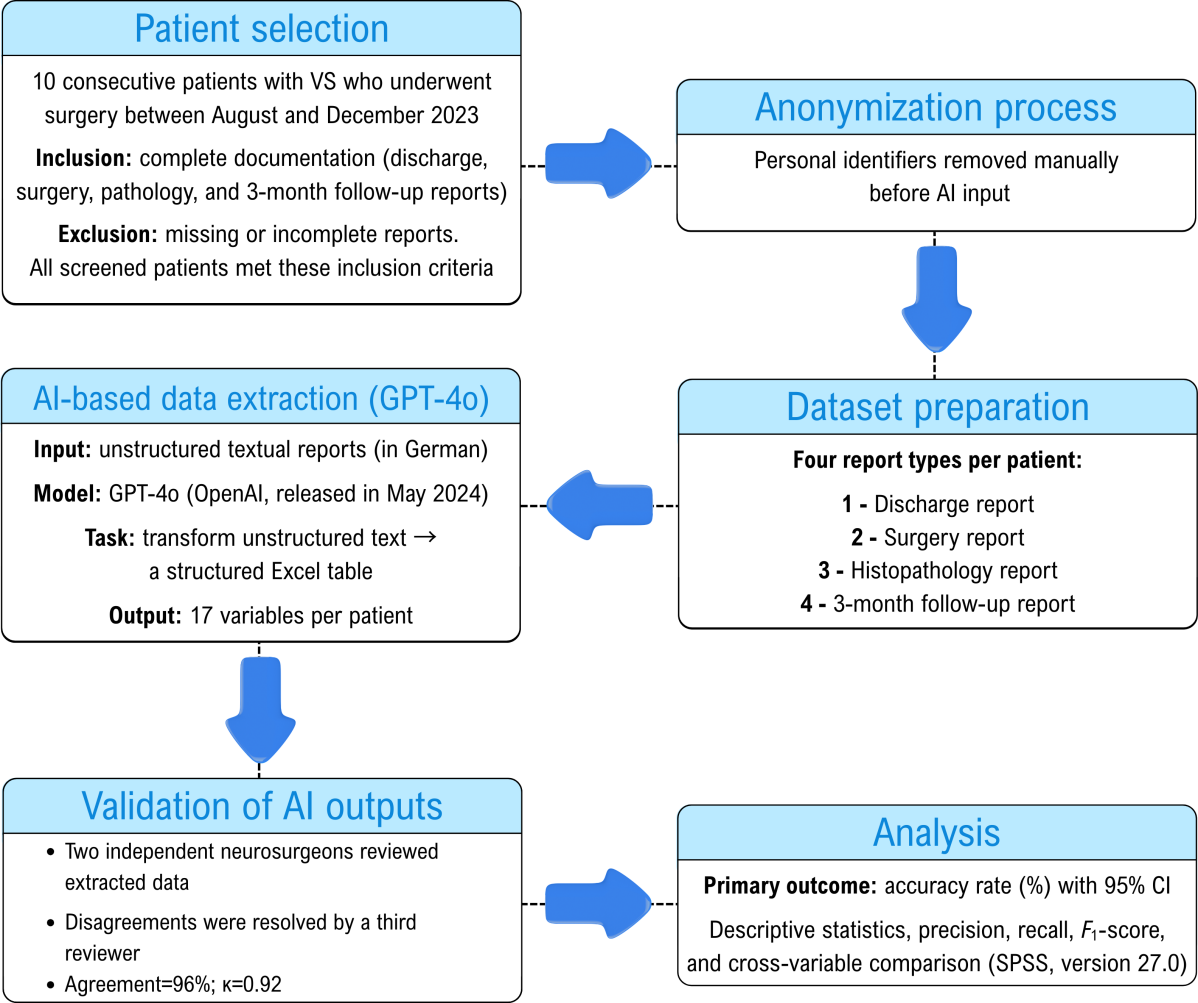
Workflow of data extraction and validation process. AI: artificial intelligence; VS: vestibular schwannoma.

This was a retrospective feasibility study conducted at a single tertiary neurosurgical center (Department of Neurosurgery at the University Hospital of Freiburg). The inclusion criteria comprised consecutive patients with histologically confirmed vestibular schwannoma who underwent surgical treatment between August and December 2023 and had complete documentation (discharge, surgical, pathology, and follow-up reports). Patients who lacked any of these reports were excluded from the analysis. Figure 1 illustrates the data flow and extraction process.

The primary outcome of the study was the accuracy rate in extracting structured information from the provided unstructured data. The ground truth for this evaluation was established by a medical team, which manually reviewed and assessed each response provided by the AI model. This ensured that the AI’s performance was rigorously compared to expert human judgment.

**Figure 2.**
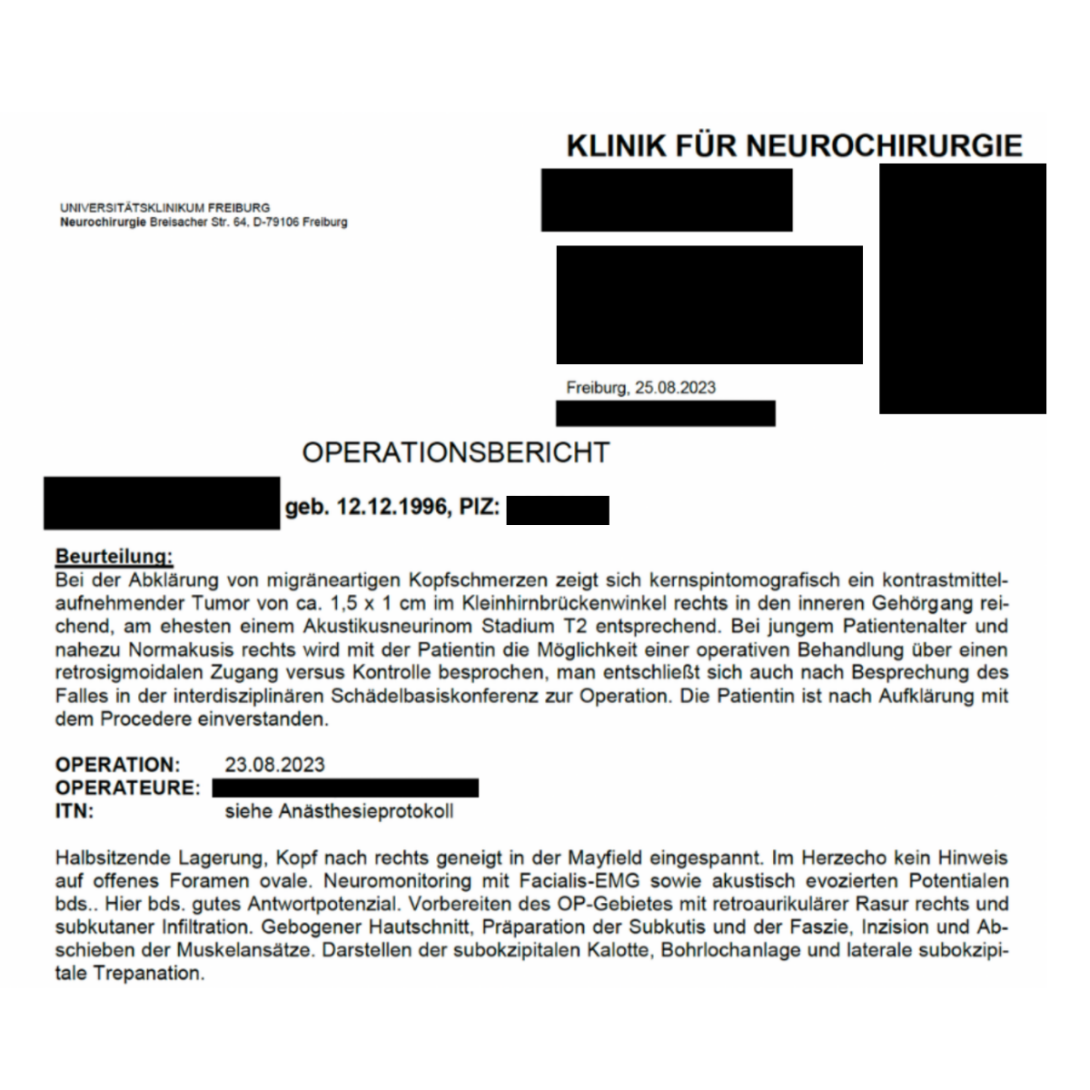
Surgical report. This surgery report was used as a raw dataset for extracting and processing unstructured data into structured data. Personal data were omitted from the artificial intelligence tool to ensure compliance with ethical standards and protect patient privacy.

All personal data of both patients and health care professionals that were visible in the original documents were anonymized before being processed by the AI model (Figures2  3). Consequently, it was not possible to identify any individual patients from the data provided to the AI model for analysis. It is also important to note that the original language of all medical reports was German, and these unstructured data were presented to the AI model in its native form for analysis and extraction.

**Figure 3.**
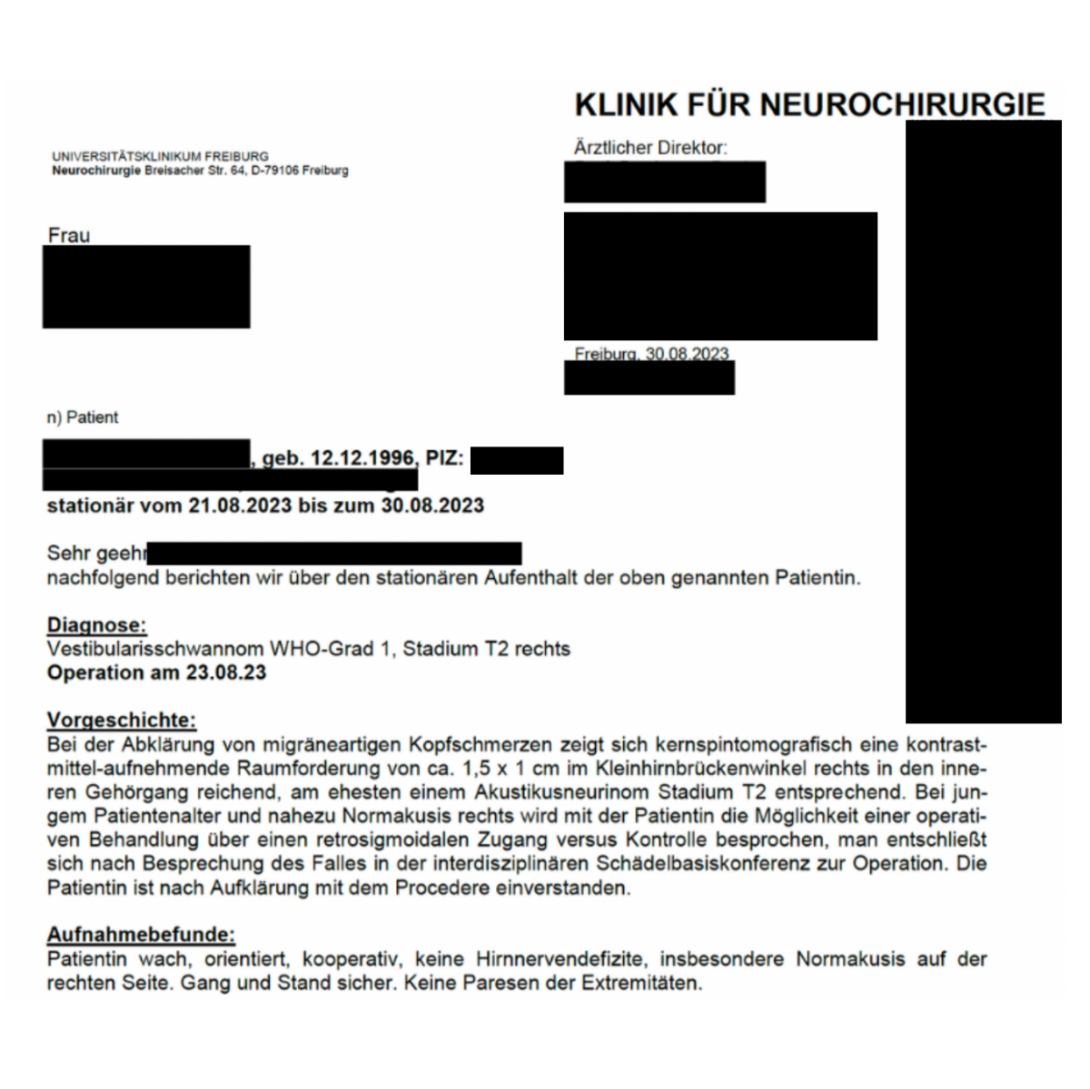
Discharge report. The discharge report was used as a raw dataset for extracting and processing unstructured data into structured data. Personal data were omitted from the artificial intelligence tool to ensure compliance with ethical standards and protect patient privacy.

### Prompt Design

Prompts were designed iteratively, meaning they were developed, tested, and refined to improve clarity and extraction precision. The initial zero-shot prompt served as a baseline framework for structured data extraction. On the basis of qualitative assessment of model responses, targeted refinements were introduced to better define certain variables that were prone to misinterpretation.

For example, the variable “intraoperative complications” was explicitly redefined in the prompt as “intraoperative damage to major vascular or neural structures,” and “new postoperative deficits” were clarified as “deficits developed after surgery, excluding preexisting ones.” These refinements were introduced to standardize semantic interpretation and reduce ambiguity during data extraction.

Refinement rounds continued until no further gains in extraction accuracy were observed, while ensuring that definitions of other variables remained unaffected. All prompts were executed in GPT-4o.

This prompt shown in Textbox 1 was used to generate a Microsoft Excel table with structured data extracted from medical reports written in natural language.

Textbox 1. **Prompt used to generate structured data table.**

I am going to upload medical reports from a few patients, and I want you to transform the textual data (unstructured) into structured data in an Excel table. The documents are: discharge report, surgery report, histopathology report, and outpatient follow-up report from 3 months post-surgery. I want you to extract data from these documents to create a structured table where each row represents a patient and each column a variable, according to the following scheme: If the information is not present in any of the provided documents, respond non applicable (n/a). The column structure is as follows:

- Column 1: PIZ (patient identification number)
- Column 2: Date of birth
- Column 3: Date of surgery
- Column 4: Symptom present at disease presentation (yes or no)
- Column 5: If yes in column 4, specify the symptom; if no, respond “n/a”
- Column 6: Time from symptom onset to surgery (in months)
- Column 7: Intraoperative complication (yes or no)
- Column 8: If yes in column 7, specify the complication; if no, respond “n/a”
- Column 9: Postoperative deficits (yes or no)
- Column 10: If yes in column 9, specify the deficit; if no, respond “n/a”
- Column 11: Was the deficit permanent? (based on the 3-month outpatient follow-up report; yes or no)
- Column 12: Histopathological diagnosis
- Column 13: WHO tumor grade
- Column 14: Tumor resection grade according to the 3-month follow-up report (1 for total resection, 2 for subtotal, 3 for recurrence)
- Column 15: Preoperative Karnofsky score (based on the admission findings in the discharge report)
- Column 16: Postoperative Karnofsky score (based on the findings upon discharge in the discharge report)
- Column 17: Any new symptoms at the 3-month follow-up visit (based on the 3-month outpatient follow-up report)

This standardized prompt was used across all patient records to ensure consistency in data extraction, with the AI tasked with transforming unstructured medical data into a format suitable for subsequent analysis.

### Data Anonymization

All original medical reports were manually anonymized by the research team before being entered into the GPT-4o interface. Identifiers such as names and addresses were removed. The anonymized reports were then processed through the official ChatGPT web platform for data extraction. No identifiable or sensitive information was transmitted, stored, or shared, ensuring compliance with General Data Protection Regulation (GDPR) and the standards of the local ethics committee.

### Data Extraction

All analyses were performed using GPT-4o accessed through the official ChatGPT interface. At the time of data collection, research API (application programming interface) access was not available. The full dataset of 10 anonymized surgical cases was uploaded as a single compressed file for structured data extraction. Generated tables were manually reviewed and archived to ensure methodological traceability and reproducibility within the proof-of-concept framework.

### Validation Process

Two board-certified neurosurgeons independently performed manual data extraction from the anonymized medical reports before the GPT-4o analysis, ensuring blinding to the AI-generated results. After completion of both manual and AI-based extractions, the datasets were compared to establish the ground truth. In cases where the 2 reviewers disagreed, a third senior neurosurgeon adjudicated the final classification. Interrater agreement between the 2 primary reviewers was calculated using Cohen κ.

### Sample Size

The sample size of 10 patients was chosen based on feasibility for this proof-of-concept design, as the objective was not to achieve statistical power but to test the model’s capacity for accurate structured data extraction in a controlled, pilot environment.

### Statistical Analysis

For each binary variable, model outputs were compared to the ground truth to derive the counts of true positives, false positives, false negatives, and true negatives. On the basis of these, accuracy, precision, recall (sensitivity), and *F*1-score were calculated primarily for binary variables, where such metrics are meaningful.

For conditional text and categorical or numerical variables, performance was mainly summarized using accuracy and corresponding 95% CIs computed via the Wilson method for binomial proportions. Additionally, Δ accuracy (postrefinement and prerefinement) was calculated to quantify improvement following prompt refinement.

To explore potential differences in accuracy across variable types—binary, conditional text, and categorical or numerical—a Kruskal-Wallis test was applied. Given the small sample size and exploratory nature of this proof-of-concept analysis, no post hoc pairwise testing or correction for multiple comparisons was performed.

All analyses were performed using SPSS Statistics (version 27.0; IBM Corp) and R software (version 4.3.2; R Foundation for Statistical Computing).

### Ethical Considerations

This research was conducted in compliance with the Code of Ethics of the World Medical Association (Declaration of Helsinki) for experiments involving human subjects. The local ethics committee of the University of Freiburg (23‐1393-S1-retro; approval date: October 31, 2023) approved the waiver of informed consent due to the retrospective design, full anonymization of patient identifiers, and minimal-risk nature of the study, consistent with institutional and national regulations. As this was a retrospective analysis of fully anonymized clinical data, no participants were contacted and no compensation was provided.

## Results

### Overview

**Figure 4.**
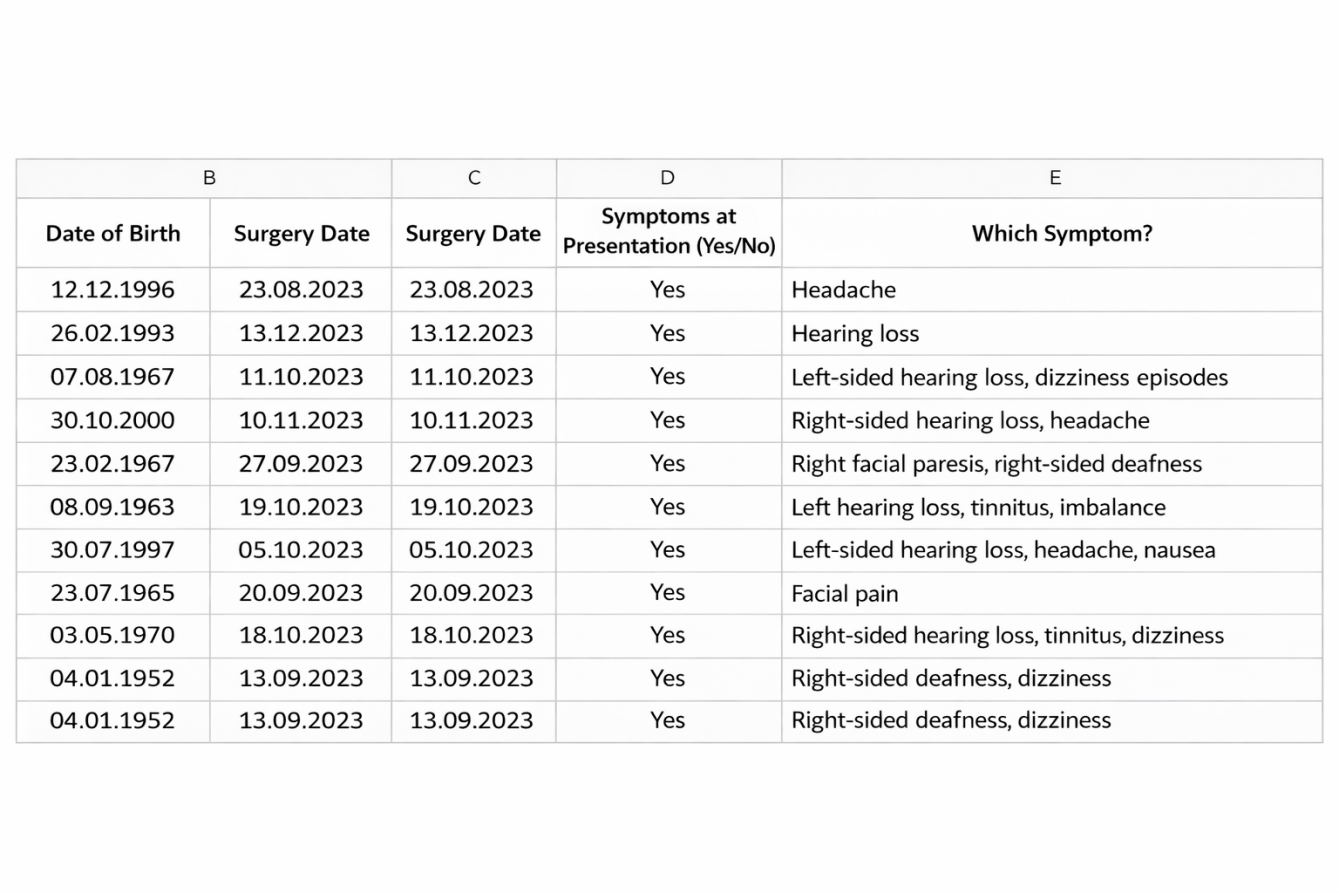
Part of the GPT-4o–generated table with structured data extracted from patient reports. These data were processed from unstructured medical records, while personal information was omitted to ensure compliance with ethical standards and protect patient privacy.

Ten consecutive patients diagnosed with intracranial acoustic neuromas who underwent surgical treatment at the Department of Neurosurgery at the University Hospital of Freiburg between August and December 2023 were included in this study. All screened patients met the inclusion criteria and were analyzed. GPT-4o generated an Excel table with structured data based on the zero-shot prompt described in the Methods section (Figure 4).

### Extraction of Structured Data

Regarding the collection of structured data that does not require text interpretation, such as date of birth, patient ID, date of surgery, histopathological diagnosis, and World Health Organization grade, and considering the physicians’ opinion as the ground truth, the accuracy rate was 100% (Table 1).

**Table 1.** Accuracy and 95% CIs (Wilson) for structured categorical variables.

Variable	Correct, n	Incorrect, n	Accuracy^a^ (%; 95% CI^b^)
Patient ID	10	0	100.0 (72.2‐100.0)
Date of birth	10	0	100.0 (72.2‐100.0)
Date of surgery	10	0	100.0 (72.2‐100.0)
Histopathological diagnosis	10	0	100.0 (72.2‐100.0)
WHO^c^ grade	10	0	100.0 (72.2‐100.0)

a All fields were extracted with perfect accuracy (100%) across 10 cases.

b CIs reflect the limited sample size rather than model variability.

c WHO: World Health Organization.

### Extraction and Processing of Unstructured Data

In the zero-shot prompt, the collection and processing of unstructured data into structured data provided heterogeneous results. The following variables presented a 100% accuracy rate when compared to the physicians’ evaluation: symptoms at presentation (yes or no), which symptoms, symptoms onset to surgery (in months), permanent deficits (yes or no), extent of resection (total, subtotal, and biopsy), and new symptoms at 3-month follow-up (Table 2).

In contrast, variables such as “intraoperative complications (yes or no),” “if yes, which complications,” “new postoperative deficits,” and “if yes, which deficits” achieved an accuracy rate as low as 50% in the zero-shot prompt. However, when the model was refined through additional prompt adjustments, as described in the Methods section, the accuracy rate improved to 90% to 100%, as presented in Tables3  4.

**Table 2.** Accuracy and 95% CIs (Wilson) for binary, categorical, and conditional text variables requiring interpretative capabilities.

Variable^a^	Correct/total, n	Accuracy (%; 95% CI)
Symptoms at presentation (yes or no)	10/10	100 (72.2-100.0)
Which symptom?	10/10	100 (72.2-100.0)
Intraoperative complication (yes or no)	5/10	50 (23.7-76.3)
Which complication?	5/10	50 (23.7-76.3)
New postoperative deficits (yes or no)	5/10	50 (23.7-76.3)
Which deficit?	5/10	50 (23.7-76.3)
Was the deficit permanent (yes or no)	10/10	100 (72.2-100.0)
Extent of resection (3-month follow-up)	10/10	100 (72.2-100.0)
New symptoms at 3-month follow-up (yes or no)	10/10	100 (72.2-100.0)

a Variables are grouped with their corresponding descriptive fields (“Which…”).

**Table 3.** Case-level model outputs for intraoperative complication before and after prompt refinement^a^.

Patient	Model performance before prompt refinement				Model performance after prompt refinement			
	Intraoperative complication	Assessment	Which complication	Assessment	Intraoperative complication	Assessment	Which complication	Assessment
1	No	Correct	None	Correct	No	Correct	None	Correct
2	Yes	Incorrect	Facial palsy (HB^b^ II)	Incorrect	No	Correct	None	Correct
3	Yes	Incorrect	Facial palsy (HB II)	Incorrect	No	Correct	None	Correct
4	No	Correct	None	Correct	No	Correct	None	Correct
5	Yes	Incorrect	Facial palsy (HB V)	Incorrect	No	Correct	None	Correct
6	Yes	Incorrect	Facial palsy (HB IV)	Incorrect	No	Correct	None	Correct
7	Yes	Incorrect	Facial palsy (HB IV)	Incorrect	No	Correct	None	Correct
8	No	Correct	None	Correct	No	Correct	None	Correct
9	No	Correct	None	Correct	No	Correct	None	Correct
10	No	Correct	None	Correct	No	Correct	None	Correct

a Each variable (“intraoperative complication” and “which complication”) was independently assessed as correct or incorrect against the ground truth. The table highlights corrections achieved through prompt refinement.

b HB: House-Brackmann.

**Table 4.** Case-level model outputs for intraoperative complication before and after prompt refinement^a^.

	Model performance before prompt refinement				Model performance after prompt refinement			
	New postoperative deficit	Assessment	Which deficit	Assessment	New postoperative deficit	Assessment	Which deficit	Assessment
1	Yes	Incorrect	Mild dizziness and mild hearing loss (right)	Incorrect	No	Correct	None	Correct
2	Yes	Correct	Mild facial paresis and left-sided hearing loss	Correct	Yes	Correct	Mild facial paresis and left-sided hearing loss	Correct
3	Yes	Correct	Left deafness, dizziness, and mild facial paresis (temporary)	Correct	Yes	Correct	Facial paresis (temporary) and left deafness	Correct
4	Yes	Incorrect	Mild right facial hypoesthesia and tinnitus	Incorrect	No	Correct	None	Correct
5	Yes	Incorrect	Facial paresis (grade V), right facial hypoesthesia, and right deafness	Incorrect	No	Correct	None	Correct
6	Yes	Incorrect	Facial paresis (grade IV), chin hypoesthesia, and left deafness	Incorrect	No	Correct	None	Correct
7	Yes	Correct	Facial paresis (grade IV) and left deafness	Correct	Yes	Correct	Facial paresis (grade IV) and left deafness	Correct
8	Yes	Correct	Facial paresis (grade V) and left deafness	Correct	Yes	Correct	Facial paresis (grade V) and left deafness	Correct
9	Yes	Correct	Mild facial paresis (right)	Correct	Yes	Correct	Mild facial paresis (right)	Correct
10	Yes	Incorrect	Mild facial paresis (HB^b^ II)	Incorrect	Yes	Incorrect	Mild facial paresis (HB II)	Incorrect

a Each variable (“New Postoperative Deficits” and “Which Deficit”) was independently assessed as correct or incorrect against the ground truth. The table highlights corrections achieved through prompt refinement.

b HB: House-Brackmann.

Model performance across interpretative binary variables is summarized in Table 5. Accuracy ranged from 50% to 100%, reflecting variable complexity in contextual interpretation. While fields such as *was the deficit permanent?* and *new symptoms at 3-month follow-up* achieved perfect accuracy, others—particularly *intraoperative complication* and *new postoperative deficits*—showed lower precision due to false-positive predictions.

**Table 5.** Model performance across interpretative binary variables.

Variable	TP^a^	FP^b^	FN^c^	TN^d^	Accuracy^e^ (%)	Precision	Recall	*F*1-score
Symptoms at presentation (yes or no)	10	0	0	0	100	1	1	1
Intraoperative complication (yes or no)	0	5	0	5	50	0	—^f^	—
New postoperative deficits? (yes or no)	5	5	0	0	50	0.5	1	0.67
Was the deficit permanent? (yes or no)	1	0	0	9	100	1	1	1
New symptoms at 3-month follow-up?	1	0	0	9	100	1	1	1

a TP: true positive.

b FP: false positive.

c FN: false negative.

d TN: true negative.

e Accuracy ranged from 50% to 100%, with lower precision observed in fields requiring finer contextual understanding, such as intraoperative and postoperative deficits.

f Not applicable.

When comparing mean accuracies across variable types using the Kruskal-Wallis test, no statistically significant difference was observed (H=2.79; *P*=.25). Nevertheless, a consistent trend toward higher accuracy for structured categorical and numerical variables (eg, *patient ID, date of birth, World Health Organization grade, extent of resection,* and *Karnofsky scores*; mean 97.5%, SD 4.6%) and lower accuracy for conditional text variables (eg, *which symptom? which complication?* and *which deficit?*; mean 66.7%, SD 28.9% supports the descriptive results. Binary variables (eg, *symptoms at presentation, intraoperative complication, new postoperative deficits, was the deficit permanent,* and *new symptoms at 3-month follow-up*) showed intermediate performance, with a mean accuracy of 80.0% (SD 27.4%).

## Discussion

### Principal Findings

The findings of this study underscore the potential of GPT-4o and similar LLMs to revolutionize health care data mining. By achieving high accuracy rates for the extraction of structured information from unstructured medical records, GPT-4o demonstrated its utility in reducing the time and effort required for data analysis. Parameters such as symptom onset, tumor grade, and extent of resection were extracted with impressive accuracy, aligning with the work of Lee et al [7], who highlighted the power of NLP tools in parsing clinical trial data efficiently. However, these findings should be interpreted within the limitations of a small, single-institution feasibility study. While they demonstrate the technical viability of GPT-4o for structured data extraction, generalization to other clinical domains requires larger, multicenter validation.

However, not all data categories achieved uniform success. Variables such as intraoperative complications and new postoperative deficits presented challenges in zero-shot scenarios, emphasizing the need for prompt refinement and iterative learning to improve model performance. Similar observations have been reported by Adamson et al [8], who noted that model tuning significantly enhances the precision of clinical data extraction from electronic health records.

Beyond these technical aspects, the integration of LLMs into clinical workflows presents transformative opportunities. For instance, these models can support operational planning by predicting the allocation of resources such as surgical supplies and human labor based on historical data trends. This capability, as highlighted by Obermeyer and Emanuel [9], could reduce inefficiencies and improve overall patient outcomes. Moreover, automated coding of diagnoses and procedures, as discussed by Dong et al [10], could streamline billing processes, thereby alleviating administrative burdens for health care institutions. It should be emphasized that this study did not assess workflow integration, time efficiency, usability, or real-time clinical applicability. Any references to potential operational or efficiency benefits are therefore speculative and reflect broader opportunities described in the literature rather than outcomes directly evaluated in this analysis.

Despite these promising applications, ethical and practical challenges must be addressed. Ensuring data privacy and security is paramount, especially when handling sensitive patient information. Adherence to privacy regulations such as the Health Insurance Portability and Accountability Act and the GDPR is critical [11,12]. Furthermore, the “black box” nature of LLMs raises concerns about their interpretability and trustworthiness in clinical settings. Transparent AI frameworks, as advocated by Arrieta et al [13], could mitigate these issues by making model predictions more explainable. In this study, model interpretability was ensured by manually auditing GPT-4o outputs and verifying their correspondence with the original clinical context. Regarding privacy, all medical reports were manually anonymized prior to being uploaded to the GPT-4o interface. The analyses were conducted using the official ChatGPT web platform, with no identifiable patient information included at any stage. No data were stored, transmitted, or processed through third-party servers outside the OpenAI environment, ensuring full compliance with institutional and GDPR standards.

Recent scholarship has underscored the ethical and operational risks of deploying LLMs in health care. Ong et al [14] highlighted how bias propagation, lack of transparency, and accountability gaps remain major barriers to safe clinical implementation. Similarly, Elbattah et al [15] emphasized the need for transparent auditing mechanisms and robust validation pipelines to mitigate privacy and safety risks in medical AI applications. In line with these perspectives, this study reinforces the necessity of rigorous data governance, responsible model deployment, and continuous ethical evaluation when integrating LLMs into clinical research workflows.

The role of NLP in augmenting documentation accuracy and quality has been demonstrated in neurosurgical contexts. For example, Sastry et al [16] revealed that NLP models improved comorbidity documentation in inpatient admissions, illustrating their potential to enhance clinical record keeping. Similarly, Biswas et al [17] explored the application of NLP in automating the detection of intraoperative elements during lumbar spine surgery, further emphasizing the model’s utility in surgical workflows. In addition, prior work has shown that LLMs can outperform manual abstraction in specific domains and significantly reduce time and labor requirements [18,19]. Compared with manual chart abstraction, which is labor intensive and time consuming, GPT-4o enabled near-instantaneous data extraction. Rule-based NLP systems, although established, require extensive customization and perform inconsistently across heterogeneous text. GPT-4o offers greater flexibility but introduces new challenges related to prompt sensitivity, hallucination risk, and limited explainability.

Another pressing concern is the potential for bias in AI models. As noted by Obermeyer et al [20], algorithms trained on biased datasets can perpetuate health care disparities, underscoring the importance of curating diverse and representative training data. Additionally, regulatory hurdles must be navigated carefully to ensure that these tools are deployed responsibly and ethically, as discussed by Char et al [21].

Looking ahead, the potential applications of LLMs in medicine are vast. Real-time decision support during surgeries, enabled by the synthesis of evidence from medical literature and intraoperative data, could significantly enhance surgical outcomes. Hashimoto et al [22] have already explored the promises and challenges of AI in surgical contexts, laying the groundwork for future innovations. Combining LLMs with complementary AI technologies, such as computer vision for imaging analysis, further expands their utility, as emphasized by Topol [23].

While GPT-4o and similar models represent a significant advancement in health care data mining, their practical implementation requires careful consideration of ethical, technical, and regulatory challenges. LLMs have the potential to not only transform data-driven research but also enhance the quality and efficiency of patient care.

This study highlights the transformative potential of LLMs such as GPT-4o in health care, particularly for mining and structuring complex medical data. The demonstrated accuracy in data extraction underscores their utility in reducing manual workloads and optimizing research and clinical processes. However, challenges such as data security, bias, and regulatory compliance remain significant hurdles. By navigating these obstacles thoughtfully, LLMs could redefine how health care systems manage and use data, ultimately improving patient outcomes and operational efficiencies. As these technologies continue to evolve, their integration must be guided by ethical frameworks and multidisciplinary collaboration to ensure their full potential is realized responsibly.

### Limitations

The small sample size represents a central limitation of this study and results in wide CIs around several accuracy estimates. Consequently, the findings should be viewed as preliminary and interpreted with caution. Although some variables reached perfect accuracy, these were predominantly straightforward structured fields requiring minimal interpretative processing. As such, these results reflect a narrow and highly controlled context and should not be extrapolated as evidence that GPT-4o performs reliably across more complex or heterogeneous clinical data extraction tasks.

Furthermore, the analysis was conducted at a single institution, and all medical reports originated from one neurosurgical service and were written in German. This linguistic and institutional homogeneity may have influenced model performance and limits the generalizability of the results to other clinical environments, documentation styles, and languages. Given the known variability in LLM performance across languages and reporting conventions, external validation is essential.

### Conclusions

Future studies involving larger, multi-institutional, and multilingual datasets, as well as more diverse clinical variables, will be necessary to assess reproducibility and determine whether these findings extend to broader clinical or operational contexts.

## Abbreviations

AI: artificial intelligence
API: application programming interface
GDPR: General Data Protection Regulation
LLM: large language model
NLP: natural language processing
